# Transcriptome profiling analysis of muscle tissue reveals potential candidate genes affecting water holding capacity in Chinese Simmental beef cattle

**DOI:** 10.1038/s41598-021-91373-2

**Published:** 2021-06-07

**Authors:** Lili Du, Tianpeng Chang, Bingxing An, Mang Liang, Xinghai Duan, Wentao Cai, Bo Zhu, Xue Gao, Yan Chen, Lingyang Xu, Lupei Zhang, Junya Li, Huijiang Gao

**Affiliations:** 1 Institute of Animal Science, Chinese Academy of Agricultural Sciences, Beijing, 100193 China; 2 College of Animal Science and Technology, Southwest University, Chongqing, 400715 China

**Keywords:** Genetics, Molecular biology

## Abstract

Water holding capacity (WHC) is an important sensory attribute that greatly influences meat quality. However, the molecular mechanism that regulates the beef WHC remains to be elucidated. In this study, the longissimus dorsi (LD) muscles of 49 Chinese Simmental beef cattle were measured for meat quality traits and subjected to RNA sequencing. WHC had significant correlation with 35 kg water loss (r = − 0.99, p < 0.01) and IMF content (r = 0.31, p < 0.05), but not with SF (r = − 0.20, p = 0.18) and pH (r = 0.11, p = 0.44). Eight individuals with the highest WHC (H-WHC) and the lowest WHC (L-WHC) were selected for transcriptome analysis. A total of 865 genes were identified as differentially expressed genes (DEGs) between two groups, of which 633 genes were up-regulated and 232 genes were down-regulated. Gene Ontology (GO) and Kyoto Encyclopedia of Genes and Genomes (KEGG) pathway enrichment revealed that DEGs were significantly enriched in 15 GO terms and 96 pathways. Additionally, based on protein–protein interaction (PPI) network, animal QTL database (QTLdb), and relevant literature, the study not only confirmed seven genes (*HSPA12A*, *HSPA13*, *PPARγ*, *MYL2*, *MYPN*, *TPI,* and *ATP2A1*) influenced WHC in accordance with previous studies, but also identified *ATP2B4*, *ACTN1*, *ITGAV*, *TGFBR1*, *THBS1*, and *TEK* as the most promising novel candidate genes affecting the WHC. These findings could offer important insight for exploring the molecular mechanism underlying the WHC trait and facilitate the improvement of beef quality.

## Introduction

Meat quality has been measured by multiple indicators such as water holding capacity (WHC), drip loss, intramuscular fat (IMF), shear force (SF), and meat color that are economically important traits with low to medium genetic heritability (*h*^*2*^)^1–5^, among which WHC is an important meat sensory attribute that contributes to improving the quality and yield of meat. Previous researches about ruminants demonstrated that extremely low WHC due to myoprotein degradation was the main cause of pale, soft, and exudative (PSE) meat, while high WHC caused by high pH could explain the production of dark, firm, and dry (DFD) meat^6^.

WHC is defined as a measurable characteristic related to the ability to retain inherent water in meat under the influence of intrinsic (i.e., genotype) and extrinsic (i.e., pre-slaughter and post-slaughter treatment methods) factors^7^. Drip loss is the most important method to assess WHC^8^. Several studies showed that the genotype played roles in the bovine WHC trait. In the work of Martínez et al., WHC was proven to exist in significant differences between diversified genotypes, which is greater in normal (+ / +) bulls, intermediate in heterozygous (mh/ +) bulls, and least in homozygous (mh/mh) bulls^9^, which was consistent with the conclusions drawn by Uytterhaegen et al. in the Belgian Blue breed^10^. Age, sex, stress, and stunning during the pre-slaughter period, as well as chilling and aging in the post-slaughter period, and meat processing methods (i.e., cooking and cooling temperature, cooking and cooling rates, etc.) all influenced the WHC^7^. Sazili et al. suggested that in comparison with cattle stunned by low power non-penetrating mechanical stunning method, those stunned by high power non-penetrating mechanical stunning method showed a lower WHC and lightness (L*)^11^. Brad Kim et al. concluded that cryogenic freezing could lead to a significant increase in WHC but decrease in SF values^12^. Additionally, WHC could directly affect other meat quality parameters, which was positively related to IMF content while negatively regulated drip loss and cooking loss^13–15^. pH was also a major element affecting the WHC^16^. Farouk et al. found WHC was higher in Bovine M. semimembranosus with inherently higher pH compared to lower pH^17^. Conversely, Wen et al. revealed WHC had significant and negative genetic correlations with pH^14^. The reason for the opposite conclusions of the above studies on the correlation between WHC and pH was that WHC was measured at different periods after animal slaughter.

In the researches of WHC, several candidate genes relevant to the trait have been identified in domestic animals. Serpin family G member 1 (*SERPING1*)^18^, cysteine and glycine-rich protein 3 (*CSRP3*)^19^, phosphorylase kinase gamma subunit (*PHKG*)^20^, ryanodine receptor 1 (*RYR1*)^15^, deiodinase, iodothyronine, type III (*DIO3*)^21^, paired-like homeodomain 2 (*PITX2*)^22^, and complement component 4 binding protein, alpha (*C4BPA*)^18^ located on SSC 2, SSC 2, SSC 3, SSC 6, SSC 7, SSC 8 and SSC 9, respectively, have been proven to be related to the WHC trait of pork. Myostatin (*MSTN*)^9^, peroxisome proliferator-activated receptor gamma (*PPARγ*)^23^, and is myopalladin (*MYPN*)^24^ mapped to BTA 2, BTA 22, and BTA 28, respectively, were identified as critical candidate genes responsible for beef WHC relying on previous studies. Besides, calpastatin (*CAST*), the specific inhibitor of the calpain family of endogenous proteases, is not only related to WHC but also correlated with tenderness in beef^25,26^. Even though amounts of genes have been identified that are related to the WHC, the gene interactions remain elusive.

The development of high-throughput RNA sequencing (RNA-seq) greatly contributes to constructing transcriptome profiling and understanding the molecular mechanisms of biological processes. However, few relevant studies on beef WHC were performed and the knowledge of molecular mechanisms underlying the trait was largely unknown. The purpose of this study is to use the RNA-Seq technique, functional enrichment tools, protein–protein interaction (PPI) network, and QTL database (QTLdb) to identify the crucial differentially expressed genes (DEGs), significant Gene Ontology (GO) terms, and Kyoto Encyclopedia of Genes and Genomes (KEGG) pathways affecting the regulation of WHC, aiming to improve the WHC trait and enhance beef quality by using molecular breeding technologies.

## Results

### Phenotypic information of Chinese Simmental beef cattle

In this study, the average of WHC, 35 kg water loss, IMF, SF, and pH for longissimus dorsi (LD) muscles of Chinese Simmental beef cattle (n = 49) was 50.00%, 36.83%, 2.47 g/100 g, 11.44 N and 5.29, respectively. The detailed summary statistics for meat quality traits were presented in Table 1. Pearson correlation coefficients between WHC and other meat traits in Table 2 showed WHC was significantly correlated with IMF (r = 0.31, p < 0.05) and 35 kg water loss (r = -0.99, p < 0.01), but not with SF (r = -0.20 p = 0.18) and pH (r = 0.11, p = 0.44). Additionally, IMF had a significant negative correlation with 35 kg water loss (r = -0.34, p < 0.05) and positive correlation with pH (r = 0.38, p < 0.01). All individuals were ranked by WHC in descending order, divided into the H-WHC group (53.10% ≤ WHC ≤ 70.11%; n = 4) and the L-WHC group (29.55% ≤ WHC ≤ 44.29%; n = 4). The average content of WHC in the H-WHC group was significantly different from that in the L-WHC group (p < 0.05), which represented the samples that could be used for RNA-seq to detect genes associated with the WHC. The detailed information of the WHC trait between the two groups was presented in Supplementary Table S1.

**Table 1:** Summary of statistical data of Chinese Simmental beef cattle quality traits.

Meat traits	N	Minimum	Maximum	Average	SD
WHC (%)	49	29.55	75.89	50.00	8.14
35 kg water loss (%)	49	17.24	52.63	36.83	6.16
IMF(g/100 g)	49	1.00	4.30	2.47	0.82
SF(N)	49	5.24	19.84	11.44	3.15
pH	49	4.76	5.77	5.29	0.19

*WHC* water holding capacity, *IMF* intramuscular fat, *SF* shear force, *SD* standard deviation.

**Table 2:** Pearson correlation coefficients between WHC and other meat traits of all samples.

Meat traits	WHC	35 kg water loss	IMF	SF	pH
WHC	1	−0.99**	0.31*	−0.20	0.11
35 kg water loss	–	1	−0.34*	0.19	−0.11
IMF	–	–	1	−0.19	0.38**
SF	–	–	–	1	−0.18
pH	–	–	–	–	1

*WHC* water holding capacity, *IMF* intramuscular fat, *SF* shear force.

**p < 0.01, *p < 0.05.

### Summary of RNA sequencing data and alignment of bovine LD muscle

The transcriptome sequencing of LD muscle tissue was conducted by RNA-seq for paired-end strategy (read length 150 bp) on an Illumina NovaSeq 6000 platform. As a result, a total of 186,968,565 raw reads, ranging from 19,721,321 to 29,214,147 for each sample were generated. After quality control, a total of 177,433,007 (an average of 22,179,126) clean reads were obtained for the eight samples, and the quality values of Q20 and Q30 were above 98.09% and 94.37%, respectively. These results indicated that the RNA sequencing quality of the samples was high. Through alignment, an average of 97.03% of clean reads was mapped to the Bos taurus reference genome, of which 90.48–92.13% and 2.71–3.75% of clean reads per sample were uniquely mappable and multiple mappable, respectively. The information on sequencing results was listed in Table 3 and Supplementary Table S2. The alignment of clean reads confirmed the reliability of the RNA-seq, which could be used for subsequent analysis.

**Table 3:** Summary of sequencing reads alignments to the *Bos taurus* reference genome.

Sample	Clean reads	Total mapped reads (%)	Uniquely mapped reads (%)	Multiple mapped reads (%)
H1	21,688,061	97.32	92.13	2.71
H2	23,789,046	97.23	91.52	3.21
H3	19,721,321	96.98	91.07	3.31
H4	21,220,022	96.86	90.98	3.18
L1	29,214,147	96.93	90.98	3.75
L2	26,307,213	96.87	90.48	3.71
L3	20,406,566	96.86	90.75	3.58
L4	24,622,189	97.16	91.79	3.28

H1, H2, H3, and H4 represent four samples of the highest WHC group; L1, L2, L3, and L4 represent four samples of the lowest WHC groups.

### Transcriptome profiling of DEGs with high and low WHC

**Figure 1:**
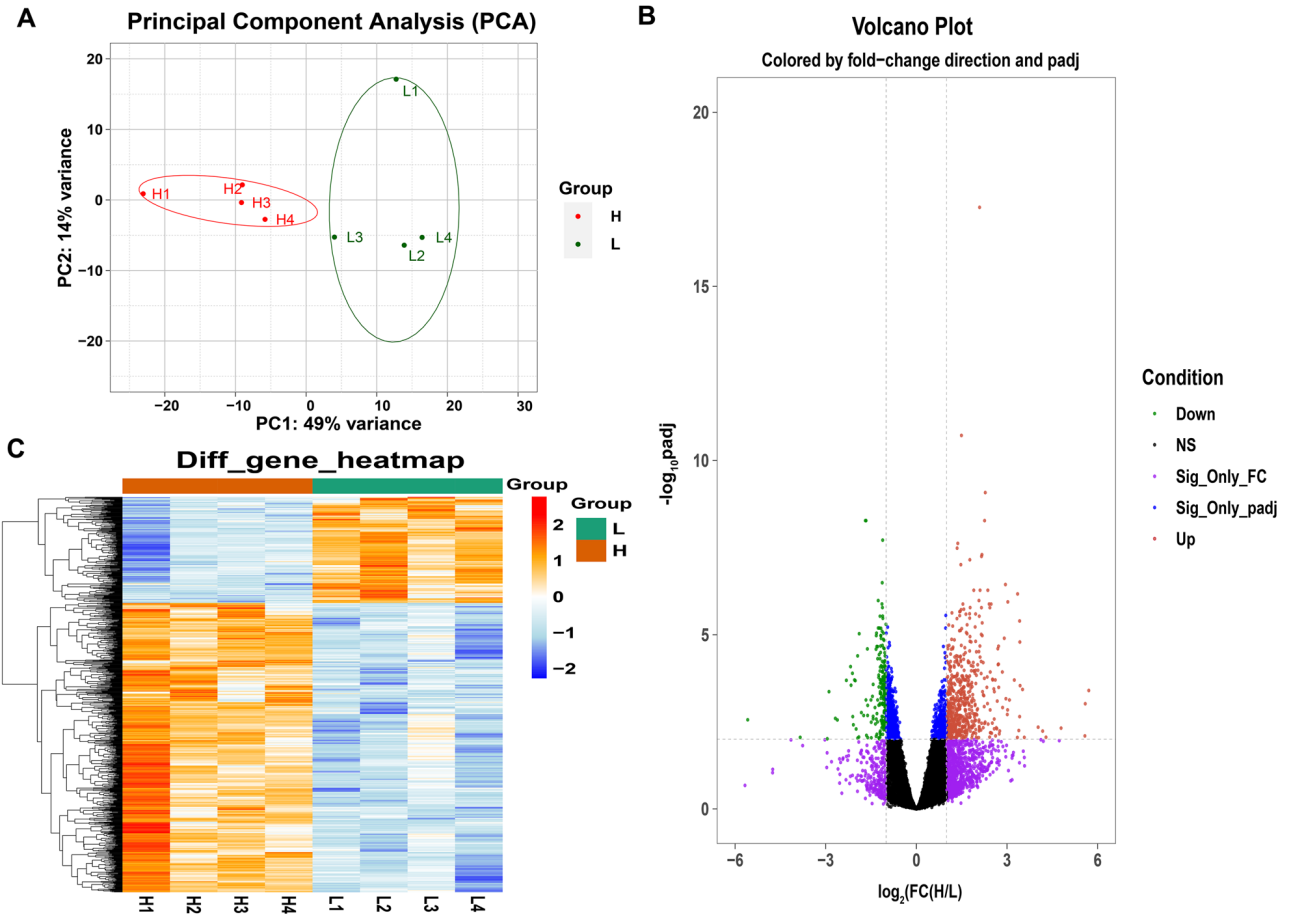
Samples correlation analysis and identification of DEGs between high WHC and low WHC groups. **(A)** PCA of the identified genes. The red and green dots represent samples of high WHC and low WHC, respectively. **(B)** Volcano plot for DEGs in LD muscle comparing high WHC group versus low WHC group. The red and green dots represent significant up-regulated (FC ≥ 2 and padj ≤ 0.01) and down-regulated (FC ≤ 0.5 and padj ≤ 0.01) DEGs, respectively. Dots of other colors indicate genes that are not significant. The purple dots denote genes with FC ≥ 2 or FC ≤ 0.5 and padj > 0.01, while the blue dots indicate genes only meet the condition of padj ≤ 0.01. The black dots represent genes with no significant change (0.5 < FC < 2 and padj > 0.01). **(C)** Heatmap of DEGs. Columns and rows show samples and DEGs, respectively. Red indicates high-level gene expression in H-WHC versus L-WHC group, while blue represents low-level gene expression in H-WHC versus L-WHC group.

The gene expression levels between H-WHC and L-WHC groups were compared to investigate the transcriptome expression profiling of the LD muscle with different WHC. Figure 1A showed two groups of individuals grouped by extreme WHC values were obviously clustered through Principal Component Analysis (PCA), which demonstrated the selection of the experimental population is reasonable. According to empirical studies, genes with a fold discovery rate (FDR) adjusted p-value less than 0.01 (padj < 0.01) and fold change ≥ 2 or fold change ≤ 0.5 (log_2_FC ≥ 1 or log_2_FC ≤ -1) were identified as DEGs. As shown in Fig. 1B, compared with the L-WHC group, a total of 865 genes were identified as DEGs in the H-WHC group, of which 633 genes were up-regulated and 232 genes were down-regulated. The results of all DEGs were displayed in Supplementary Table S3. Furthermore, Fig. 1C indicated the hierarchical clustering of heatmap depended on all DEGs was consistent with PCA analysis. Red and blue indicated the high-level and low-level gene expression in the H-WHC group versus the L-WHC group, respectively, which showed the gene expression patterns were consistent within groups and different between groups.

### GO and KEGG pathway enrichment analyses

**Figure 2:**
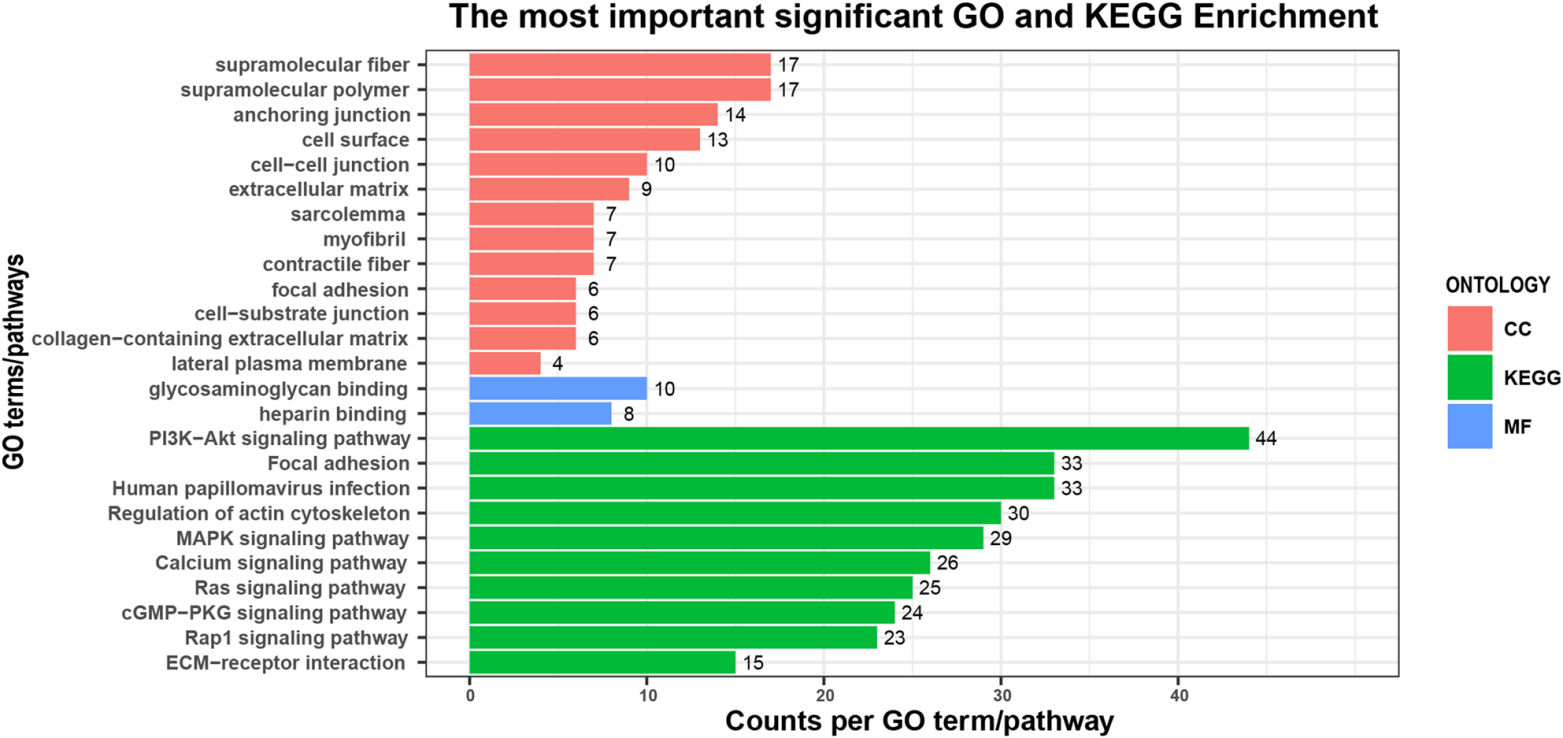
GO terms and KEGG pathways analyses of all DEGs between H-WHC and L-WHC groups. The x-axis and y-axis represent the number of DEGs enriched per GO term or KEGG pathway, and the most highly enriched GO terms or pathways, respectively. The numbers in the figure represent the number of DEGs enriched to each GO term or pathway.

GO and KEGG enrichment analyses were performed to further understand the function of the DEGs. Figure 2 showed the significantly enriched GO terms and pathways (p-value < 0.05 and q-value < 0.05). A total of 15 significant GO terms were enriched, among which 13 terms were involved in the cellular component (CC) category (cell surface, extracellular matrix, and focal adhesion, etc.), two terms were enriched in the molecular function (MF) category (heparin binding and glycosaminoglycan binding), but none of the terms participated in biological process (BP) category. As shown in Table 4, among these GO terms, DEGs were mainly enriched in the cell surface, anchoring junction, extracellular matrix, and sarcolemma, implying that these biological processes might play crucial roles in the WHC trait. Figure 2 and Table 4 also displayed some significantly enriched pathways that were mainly associated with environmental information processing, such as ECM-receptor interaction (bta04512), the mitogen-activated protein kinase (MAPK) signaling pathway (bta04010), etc. And three pathways were classified into cellular processes, including focal adhesion (bta04510), regulation of actin cytoskeleton (bta04810), and adherens junction (bta04520). Most of the pathways were associated with signal transduction, cellular processes (cell growth, cell proliferation, cell division, and cell differentiation), and muscle development. The detailed information about significant GO terms and pathways was shown in Supplementary Table S4 and Supplementary Table S5.

**Table 4:** Most important GO terms and pathways of DEGs between H-WHC and L-WHC groups.

ID	GO term/pathway	p-value	Number of genes	Key genes
GO:0009986	Cell surface	9.22E−05	13 (3)	*THBS1/TGFBR1/ TEK*
GO:0070161	Anchoring junction	1.54E−04	14 (4)	*ACTN1/ITGAV/TGFBR1/TEK*
GO:0031012	Extracellular matrix	3.98E−04	9 (1)	*THBS1*
GO:0042383	Sarcolemma	4.66E−04	7 (2)	*ACTN1/ATP2B4*
GO:0030016	Myofibril	1.04E−03	7 (3)	*ACTN1/ATP2B4/MYL2*
GO:0043292	Contractile fiber	1.38E−03	7 (3)	*ACTN1/ATP2B4/MYL2*
GO:0005925	Focal adhension	1.75E−03	6 (3)	*ACTN1/ITGAV/TEK*
GO:0005911	Cell–cell junction	2.04E−03	10 (3)	*ACTN1/TGFBR1/TEK*
bta04510	Focal adhesion	1.50E−13	33 (4)	*ACTN1/ITGAV/THBS1/ MYL2*
bta04810	Regulation of actin cytoskeleton	1.76E−10	30 (3)	*ACTN1/ITGAV/ MYL2*
bta04512	ECM-receptor interaction	5.55E−07	15 (2)	*ITGAV/ THBS1*
bta04520	Adherens junction	8.61E−04	9 (2)	*ACTN1/TGFBR1*
bta04010	MAPK signaling pathway	6.42E−07	29 (2)	*TGFBR1/TEK*

*GO* gene ontology, *KEGG* Kyoto Encyclopedia of Genes and Genomes, *DEGs* differently expressed genes, *MAPK* mitogen-activated protein kinase, *ECM* extracellular matrix.

Number of genes: the first number represents the total number of genes enriched per GO term or pathway; the second number represents the number of key genes displayed in the next column. Combined with the biological function analysis of genes and the previous relevant studies on the regulatory mechanism of WHC, the DEGs involved in more than three GO terms and three pathways could be identified as key genes that are listed in the fifth column of the table.

**Figure 3:**
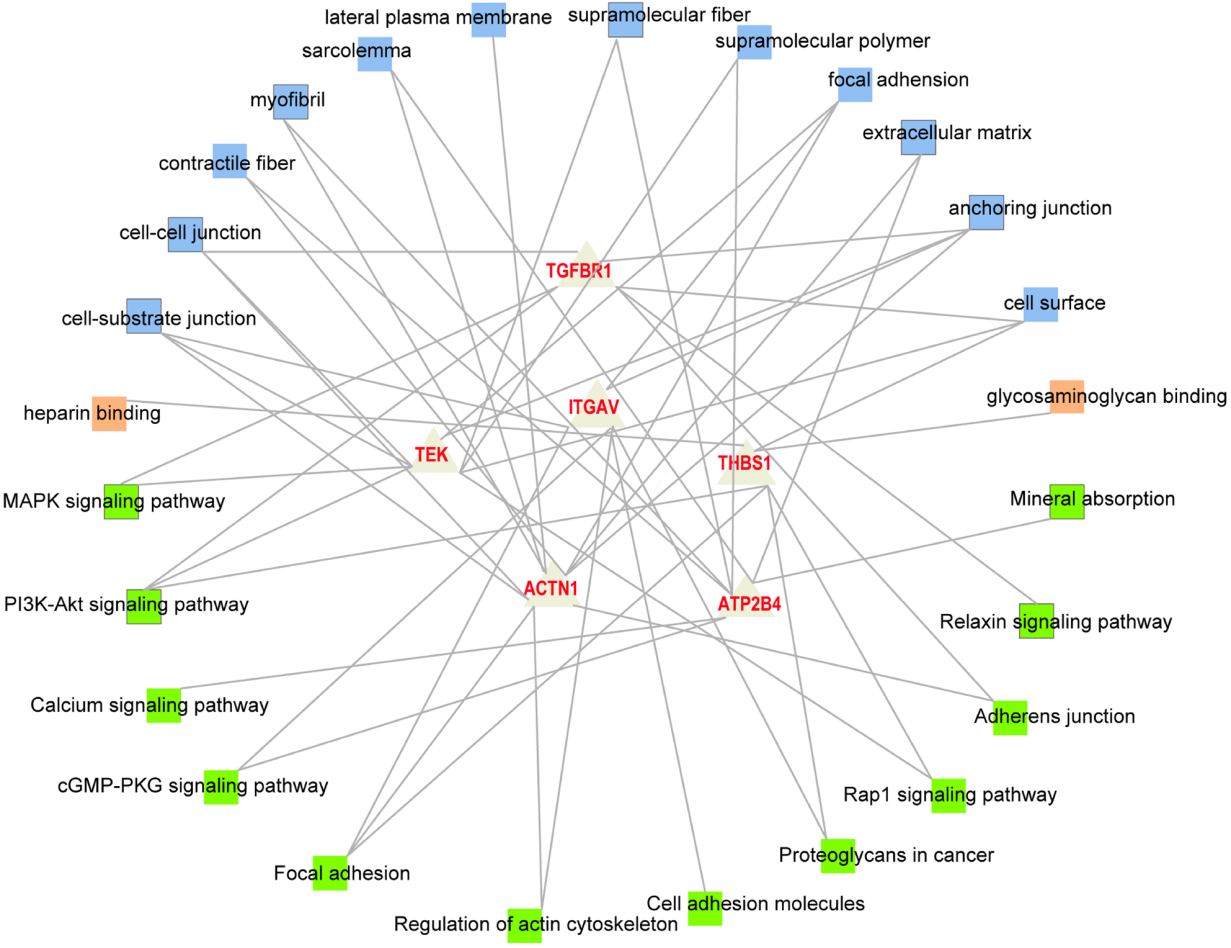
The network diagram of the novel candidate genes affecting the WHC and their belonged GO terms and pathways. Blue and orange squares represent the enriched GO terms. Green squares represent the enriched pathways.

Figure 3 showed the network diagram where the novel candidate genes were significantly enriched in some GO terms and pathways. Combined with the biological function analysis of genes and previous studies on the regulatory mechanism of WHC, the DEGs associated with more than three GO terms and three pathways could be recognized as potential candidate genes associated with WHC. Consequently, *ATP2B4*, *ACTN3*, *ITGAV*, *TGFBR1*, *THBS1*, and *TEK* were identified as novel potential candidate genes regulating WHC following the transcriptome analysis. Table 5 showed the information of these six genes.

**Table 5:** Six potential candidate genes affecting the WHC between two groups.

Symbol	BTA	log_2_FC	padj	Gene position (bp)	Gene description
*ATP2B4*	16	1.65	5.39E−03	1,400,155–1,497,069	ATPase plasma membrane Ca^2+^ transporting 4
*ACTN1*	10	1.25	9.40E−04	80,672,883–80,775,228	Actinin alpha 1
*ITGAV*	2	1.47	5.83E−05	9,644,368–9,749,556	Integrin subunit alpha V
*TGFBR1*	8	1.23	3.12E−03	64,107,418–64,179,245	Transforming growth factor beta receptor 1
*THBS1*	10	2.28	8.21E−10	35,209,595–35,224,867	Thrombospondin 1
*TEK*	8	1.29	3.93E−03	64,107,418–64,179,245	Transforming growth factor beta receptor 1

*WHC* water holding capacity, *BTA* *Bos taurus* autosome, *FC* fold change, *padj* p-value adjusted by false discovery rate (FDR).

Gene position (bp): position (bp) on ARS-UCD1.2.

### Screening DEGs based on QTLdb and previous reports

To further search for candidate genes affecting WHC, we analyzed the DEGs in the cattle QTLdb (https://www.animalgenome.org/cgi-bin/QTLdb/BT/index). Quantitative Trait Locus (QTLs) for drip loss or WHC have been found on BTA 1, 2, 4, 7, 11, 14, 19, 22, 28, and 29. However, genes influencing the WHC or drip loss identified in these QTLs remain still very limited. As listed in Supplementary Table S6, only a total of 15 QTLs in the cattle QTL database were reported to be associated with WHC and drip loss, which indicated a lack of researches on cattle WHC. Besides, Table 6 showed several genes affecting the WHC reported by previous studies. Consistent with previous studies, *HSPA12A*, *HSPA13*, *PPARγ*, *MYL2*, *MYPN, TPI1*, and *ATP2A1* were identified in this study and these genes might be involved in the WHC trait. Notably, *PPARγ*, *MYPN*, and *ATP2A1* were differently expressed in the two groups only when padj < 0.05. The information of these genes could be searched in Supplementary Table S3 and Supplementary Table S7.

**Table 6:** Candidate genes related to WHC or drip loss reported in previous reports.

Symbol	BTA	Gene position (bp)	GC content (%)	References
*HSPA1L*	23	27,523,225–27,527,209	45.55	Reference^64^
*HSPB1*	25	34,345,339–34,347,009	67.44	Reference^64^
*HSPB7*	2	136,053,155–136,057,934	64.67	Reference^64^
*HSPH1*	12	29,796,159–29,819,628	39.42	Reference^64^
*PPARγ*	22	56,709,248–56,835,386	41.26	Reference^23,81^
*MYH1*	19	29,483,027–29,507,056	41.84	Reference^71^
*MYH7*	10	21,325,414–21,345,624	55.05	Reference^98^
*MYH10*	19	28,063,029–28,183,409	44.00	Reference^71^
*MYL2*	17	54,706,765–54,714,580	44.93	Reference^85^
*MYPN*	28	24,679,611–24,593,260	39.69	Reference^24^
*MSTN*	2	3,631,373–3,851,228	34.05	Reference^9^
*TPI1*	5	103,580,087–103,583,951	60.26	Reference^89^
*ACTN3*	29	44,582,264- 44,596,714	59.26	Reference^99^
*ATP2A1*	25	25,929,240–25,946,430	55.92	Reference^49^

*BTA* *Bos taurus* autosome.

Gene position (bp): position (bp) on ARS-UCD1.2.

### PPI analysis of candidate genes

**Figure 4:**
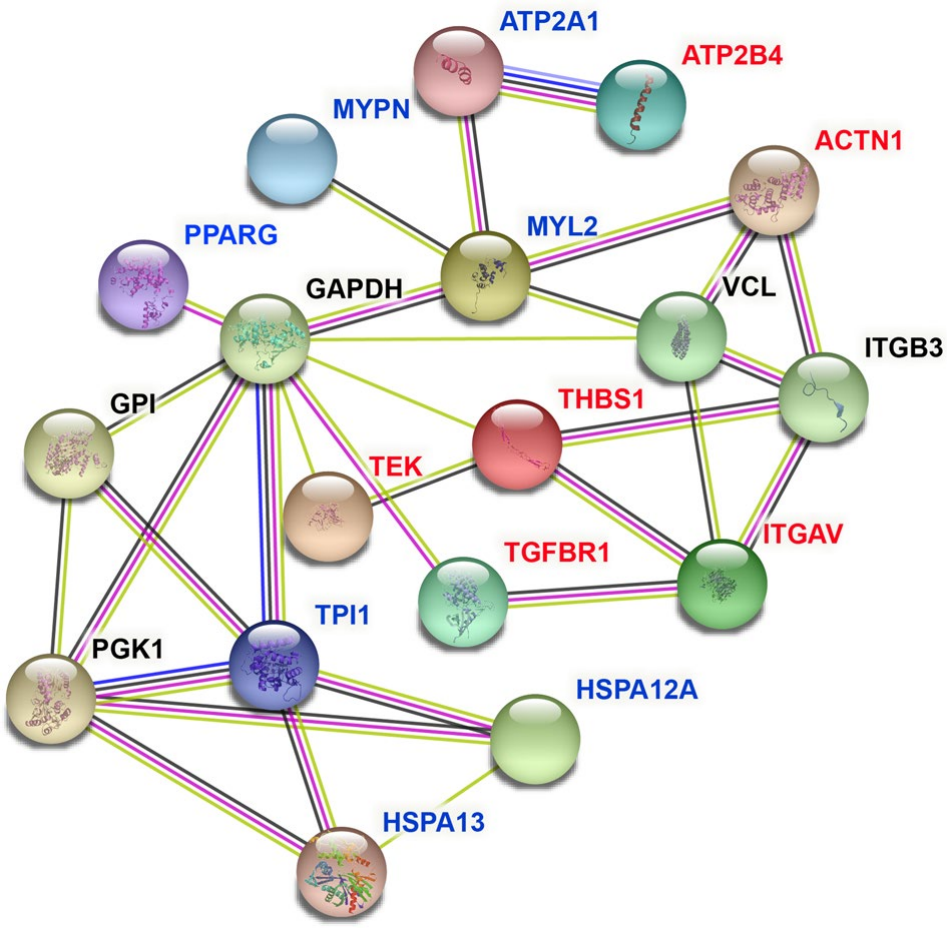
PPI network of the candidate genes affecting the WHC. The novel potential candidate genes influencing WHC found in this experiment are marked in red, while genes marked in blue represent they have been confirmed by predecessors to be related to WHC.

To visualize the interaction between node proteins encoded by potential candidate genes, we used Search Tool for the Retrieval of Interacting Genes (STRING) for PPI network analysis, which was shown in Fig. 4. The novel potential candidate genes identified in this experiment that influenced WHC were marked in red and genes that had been confirmed by previous studies to be related to WHC were marked in blue. The detailed information of these genes was listed in Supplementary Table S7 and the involvement of these genes in GO terms and pathways were presented in Supplementary Table S8.

## Discussion

WHC was an important sensory attribute that could directly affect other meat quality traits. Previous studies indicated WHC was positively related to IMF while negatively regulated drip loss^13,15^. In this study, WHC had a positive correlation with IMF, which coincided with the conclusions reported by Bhuiyan et al.^5^, Jung et al.^13^, and Watanabe et al.^27^. In addition, WHC and IMF were negatively correlated with SF although the results were statistically nonsignificant. Derington et al. showed IMF content was negatively related to SF^28^, which had been confirmed by Ueda et al. in Japanese Black steer^29^. Nevertheless, Ling et al. pointed the increase of IMF content from 7.7 to 17.4% had no significant effect on SF^30^. Compared with IMF, pH was more suitable as an important factor influencing WHC^27^. In this study, pH had a significant correlation with IMF, but not with WHC, which was consistent with the correlation between pH and IMF reported in the previous study^27^. WHC increased linearly as pH increased in the LD muscle of beef^31^, inversely, it was negatively correlated with pH in pork and partridge^14,32^. The relationship between pH and WHC was not consistent among studies, including our own.

**Figure 5:**
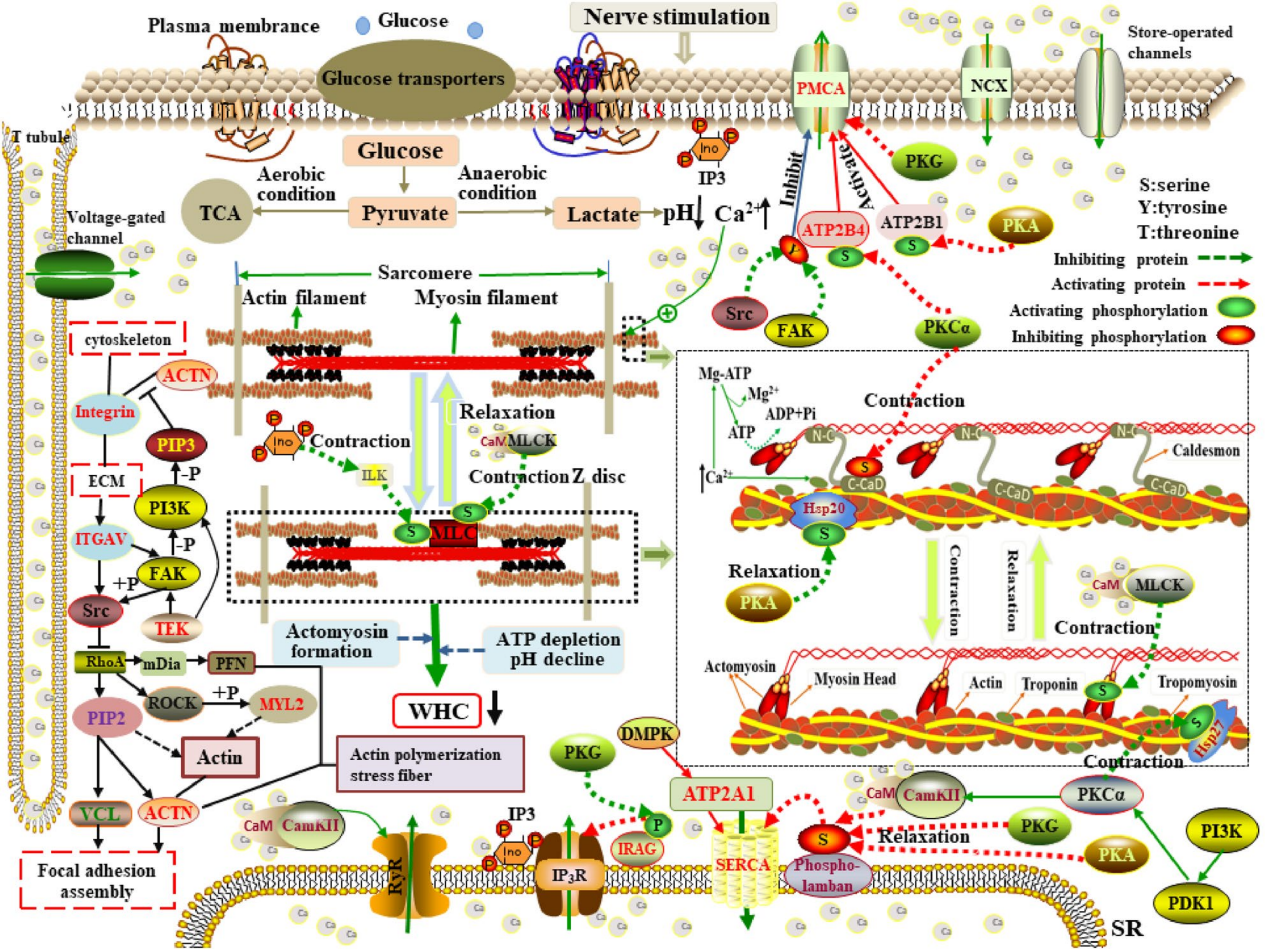
The mechanism of WHC variation in skeletal muscle contraction and relaxation. When the muscles receive external stimulation, the influx of external Ca^2+^ via channels induces the depolarization of the sarcolemma, thus resulting in the following events. (1) The depolarization of transverse tubules (T tubules) allows cytoplasmic Ca^2+^ to be released into the sarcoplasm. (2) The depolarization is transmitted via the T tubules to the SR and acts on protein complexes such as ryanodine receptors and inositol triphosphate (IP3) receptors in the SR, contributing to the release of Ca^2+^ from the SR into the sarcoplasm. (3) Ca^2+^ binds to the troponin C subunit (TnC) and then induces the tropomyosin to shift deeper into the grooves of the actin. Exposure of actin active sites allows for myosin head binding. Concurrently, the release of ATP from the inert Mg-ATP complex activates the myosin head ATPase. (4) Myosin binds with actin to form contractile actomyosin. Contraction of myofibrils ultimately leads to the movement of water out of the muscle cell into the extracellular space. (5) When the action potential disappears, Ca^2+^ is extruded to the extracellular space by the NCX and the PMCA and transported to the SR via the SERCA. Actomyosin is dissociated due to the recombination of tropomyosin and actin caused by the separation of Ca^2+^ and troponin, which allows for expansion of the myofibril and makes more room for water.

As shown in Fig. 5, the retention and loss of water in the muscle are extremely governed by its swelling and shrinkage^33^. Postmortem glycolysis under anaerobic conditions causes pH value to drop to the pI in response to the upsurge of lactic acid and results in the inefficient generation of adenosine triphosphate (ATP). In the absence of ATP, actomyosin is unable to be broken, leading to stiffness in the muscle known as rigor mortis. The dramatic decrease in WHC during rigor mortis is due to muscle contraction caused by pH decline and the depletion of ATP, which leads to the release of Ca^2+^ from the sarcoplasmic reticulum (SR) into sarcoplasm and the reduction of the space between the myosin and actin, ultimately expelling more water from the myofibril^34^. However, fasting for at least 24 h, feeding low-starch diets, and the injection of adrenaline and insulin before slaughter, as well as carcass chilling, electrical stimulation within 45 min, and the addition of salt (sodium chloride, diphosphate, pyrophosphate, etc.) after slaughter could be performed to curtail postmortem anaerobic glycolysis and pH decline, thus increasing WHC and improving meat quality^7^. In the present study, we identified several novel potential candidate genes significantly enriched in more than three GO terms and three pathways were likely to regulate the WHC. The ionized calcium (Ca^2+^) homeostasis is tightly regulated by many elements, such as the plasma membrane Ca^2+^ transport ATPases (PMCA), Na^+^/Ca^2+^ exchanger (NCX), and sarco/endoplasmic reticulum Ca^2+^-ATPase (SERCA)^35,36^. PMCA isoforms 4 (ATP2B4, aka PMCA4), encoding by the *ATP2B4* gene, is mainly responsible for transporting excess Ca^2+^ through the plasma membrane to fine-tune the cytosolic Ca^2+^ concentration^37^. The PMCA4 active sites are located in between the 4th and 5th transmembrane domains and its long C-terminal region contains the calmodulin-binding domain (CBD)^38^. PMCA4 activity is positively regulated by high Ca^2+^ concentration through the interaction with Ca^2+^-CaM complex and CBD^39^, the involvement of phosphoinositol-4,5-bisphosphate (PIP2) by improving PMCA4 affinity to Ca^2+^^40^, and phosphorylation of PMCA4 serine/threonine residues induced by protein kinase (PKA, PKG and PKC)^41,42^, whereas negatively correlated with phosphorylation of PMCA4 tyrosine residues mediated by Src kinase^43^. Activated PMCA4 couples the transport of Ca^2+^ out of the intracellular environment to regulate muscle relaxation and contraction. However, under severe stress conditions, *ATP2B4* is down-regulated that decreases the extrusion of cytoplasmic Ca^2+^ while increases the sarcoplasmic Ca^2+^ concentration, which triggers muscle contraction and then expels more water from the cells. The sarcomere length in the contractile state of muscle is shorter than that in the normal state, and WHC decreases with decreasing sarcomere length^44,45^. Consequently, the involvement in Ca^2+^ extrusion and myofibrillar relaxation/contraction of *ATP2B4* indicates it affects the WHC. Contrary to the PMCA4, sarco/endoplasmic reticulum calcium ATPase 1 (ATP2A1, aka SERCA1), encoded by *ATP2A1*, is the main regulator for the reuptake of cytosolic Ca^2+^ into the SR. SERCA1 contains four transmembrane helices that are associated with Ca^2+^ binding and translocation^46^. The missplicing of *SERCA* could affect the regulation of Ca^2+^ concentration of the SR and lead to excessive contraction^47^, and mutations in *ATP2A1* resulted in abnormalities of Ca^2+^ transmembrane flux, which could account for the muscle stiffness^48^. As mentioned previously, muscle stiffness has detrimental effects on WHC. Ciobanu et al. had reported *ATP2A1* was the candidate gene regulating WHC^49^. Therefore, *ATP2A1* can be recognized as the candidate gene regulating WHC. Alpha-actinin (α-actinin), as the primary z-disk protein, interacts with many other proteins like integrins, vinculin, and talin to mediate the linkage of actin filaments for focal adhesion, sarcomere function, and cell adhesion^50^. Alpha-actinin 1 (ACTN1) encoded by the *ACTN1* gene can bind actin in the cytoskeleton to coordinate cell adhesion through regulation of focal adhesion kinase-Src interaction^51^. Given the evidence suggesting that *ACTN1* is downregulated during normal myoblast differentiation^52^. Notably, in this study, *ACTN1* was significantly and differently expressed in the two groups. Increased expression of *ACTN1* can stimulate cell migration and reorganize the actin cytoskeleton^53^. As the direct substrate for focal adhesion kinase (FAK), α-actinin is involved in FAK-dependent signals that influences the formation of focal adhesion and the linkage between integrin and cytoskeleton^54^. Focal adhesions (FAs) formed in the absence of α-actinin reduce its adhesiveness to the extracellular matrix (ECM). The phosphorylation of ACTN1 at tyrosine-12 (Y12) induced by FAK can reduce its binding affinity to actin, whereas contributes to stress fiber formation and focal adhesion maturation^55^. Simultaneously, the activation of phosphatidylinositol 3-kinase (PI3K) catalyzes a substrate to produce phosphatidylinositol-3,4,5-triphosphate (PIP3). The binding of PIP3 to ACTN1 interrupts its interaction with the integrin β subunit^56^, as well as it enhances the hydrolysis of ACTN1 by protease and then destroys the binding of α-actinin to actin filaments^57^, which leads to the promotion of cytoskeleton flow. Unlike PIP3, PIP2 stabilizes ACTN1 junctions structure^57^. ACTN1 belongs to the calcium-sensitive α-actinin^58^. Drmota et al. proposed Ca^2+^ has negatively regulated the activity of ACTN1, leading to impaired ability of F-actin cross-linking protein^59^. In addition, ACTN1 interacts with the α-subunit of Ca^2+^ calmodulin-dependent protein kinase II (CaMKII) and other molecules to affect the Ca^2+^ pump in the plasma membrane^60^. Hence, *ACTN1* may be considered as the important candidate gene for WHC as it regulates cytoskeleton morphology and F-actin cross-linking protein. Integrin alpha-V (*ITGAV*), as a member of the integrin family, plays a critical role in the attachment of the cytoskeleton to the ECM^61^. Reports showed that postmortem degradation of integrin contributed to the formation of drip channels^62^, decreasing the ability of the water retention in the muscle^63^. Alterations in the architecture of myofibrils have an impact on the water-retaining properties of muscle cells^64^, thus the pathway of “regulation of actin cytoskeleton” is recognized as the most potential candidate pathway affecting WHC^65^. *ITGAV* is involved in this pathway in the present study. Besides, thrombospondin-1 (*THBS1*) encodes the ECM adhesive glycoprotein and binds to *ITGAV* to regulate focal adhesion disassembly and cell-to-matrix interactions^66^, which was significantly enriched in extracellular matrix (GO:0031012), focal adhesion (bta04510), and ECM-receptor interaction (bta04512) in this study. ECM contains many proteins such as glycoproteins, proteoglycans, and collagens that affect meat quality greatly like increasing WHC and regulating the tenderness^67,68^. In terms of adhesion, the best-characterized aspect is muscle connection with other muscles may require an integrin-mediated linkage between the ECM and the actin cytoskeleton. Drip loss can be decreased due to the separation of the ECM from the cytoskeleton^69^. These findings suggest *ITGAV* can interact with *THBS1* to be involved in the regulation of WHC by affecting cytoskeleton, EMC and focal adhesion . Transforming growth factor-beta receptor 1 (*TGFBR1*) was significantly enriched in three GO terms and 13 pathways. *TGFBR1* plays an important role in the synthesis of cadherin, skeletal muscle development and TGF-β signal transduction^70^. Muscle fibers are the main composition of skeletal muscle, whose development is closely associated with meat quality traits in livestock such as WHC^71^, IMF^72^, and tenderness^73^. TGF-β signaling is involved in the ECM formation and remodeling^74^. ECM plays roles not only in the integrity and growth of skeletal muscle, but also in the adaptation of myofibrillar structures and signal transduction from the ECM to the myoblast^75^. Therefore, biological function and pathways analyses of this gene reveal that it plays a potential role in the WHC. Angiopoietin-1 receptor (TEK) encoded by *TEK* gene participates in plenty of biological functions, such as regulating the reorganization of the actin cytoskeleton and focal adhesion assembly. In this study, *TEK* is significantly enriched in seven GO terms including focal adhesion, anchoring junction and cell surface, and four signal transduction pathways that contains the MAPK signaling pathway. FAs combine the actin cytoskeleton with the ECM, and amounts of intracellular signals are transmitted by FAs^76^. In the pathway of “focal adhesion”, ANGPT1 oligomers recruit TEK to form complexes and combine with TEK molecules from adjacent cells, which leads to the preferential activation of PI3K, as well as TEK can promote the activation of FAK. Under the co-regulation of PI3K and FAK, the production of PIP3 destroys the binding of α-actinin to actin filaments, which ultimately promotes cytoskeleton flow and the changes in cytoskeleton morphology affect WHC. *TEK* affects the formation of supramolecular fiber and thus it is closely associated with WHC and drip loss^77^. Consequently, its involvement in biological processes and signal transduction indicates that it affects the development of WHC.

In addition to the novel candidate genes mentioned above, we also confirmed several genes regulating the WHC reported in the previous studies. Heat shock protein 70 (HSP70) was involved in WHC due to it could protect proteins from denaturing caused by lethal heat shock^78^. And the improvement of these proteins' abundance could contribute to less fluid exuding from the cells^79^. Zhao et al. reported *HSPA1L*, *HSPB1*, *HSPB7*, and *HSPH1* were related to drip loss^64^. In this study, heat shock protein family A (HSP70) member 13 (*HSPA13*) and heat shock protein family A (HSP70) member 12A (*HSPA12A*) were differently expressed between the H-WHC and L-WHC groups. Peroxisome proliferator-activated receptor gamma (*PPARγ*) is a ligand-activated nuclear hormone receptor subfamily of transcription factors that regulates glucose homeostasis^80^. The mutations of the CDS region in *PPARγ* have a potential correlation with WHC and tenderness^81^. Overall, it can be concluded that *HSPA13*, *HSPA12A*, and *PPARγ* play an important role in beef WHC. Most of the water is stored in myofibrils^82^, and the denaturation of myofibrillar proteins is closely associated with low WHC^64^. Myosin is the most abundant of myofibrillar proteins that affect the development of bovine skeletal muscles^83^, which is composed of heavy (MHC) and light (MLC) chains^84^. In PSE pork, myosin denaturation leads to myofibrillar shrinkage, thus resulting in high drip loss^82^. MYL family genes have been identified as potential candidate genes for WHC prediction in the research of yak muscle^85^. In the present study, one myosin light chain family gene (*MYL2*) was significantly enriched in four GO terms and seven pathways. The above shows *MYL2* may be a potential candidate gene regulating WHC. Myopalladin (*MYPN*) is an encoding gene of the sarcomere protein that regulates Z-line and I-band protein assemblies^86^. As discussed previously, WHC changes with the variation in sarcomere length^44^. *MYPN* was an important candidate gene for meat quality selection^87^, which could regulate WHC in cattle breeding^24^. Although *MYPN* was differentially expressed only when padj < 0.05 in this experiment, it could also be conjectured that *MYPN* was the candidate gene that affected the WHC. Experiments have shown that denaturation of sarcoplasmic proteins played a special role in WHC reduction^88^. Triosephosphate isomerase (*TPI1*) encodes triosephosphate isomerase that belongs to sarcoplasmic protein, which has been identified as the potential candidate gene related to beef meat quality like WHC^89^, drip loss^90^, and ultimate pH^91^. These results indicate that *TPI1* may responsible for the development of WHC.

In conclusion, this study revealed the correlation between WHC and other meat attributes, indicating WHC was an important indicator to reflect meat quality. Based on transcriptome analysis as well as the integration of GO and pathway enrichment, PPI, and previous relevant studies, several novel potential candidate genes and pathways were identified to be involved in the WHC mainly by regulating the concentration of Ca^2+^ in sarcoplasm, influencing the binding of actin to myosin, and affecting the synthesis, degradation, and denaturation of the specific proteins including integrin, myofibrillar protein, sarcomere protein, and sarcoplasmic protein. These findings will provide effective references for exploring the molecular mechanism of beef WHC and contribute to improving meat quality.

## Methods

### Ethics declarations

The study was approved by the Ethics Committee of Science Research Department of the Institute of Animal Science, Chinese Academy of Agricultural Sciences (CAAS), Beijing, China (approval number: RNL 09/07). All the animal procedures were not only performed strictly according to the guidelines proposed by the China Council on Animal Care and the Ministry of Agriculture People’s Republic of China but also in compliance with the Animal Research: Reporting In Vivo Experiments (ARRIVE) guidelines. The use of animals and private land in this study was approved by their respective legal owners.

### Animals and sample collection

A total of 49 Chinese Simmental beef bulls with an average age of 26 months and an average pre-slaughter weight of 700 kg were obtained to eliminate the influence of farm, age, and sex differences on the results of the LD muscle transcriptome, among which eight Chinese Simmental beef bulls that came from different sires and dams were subjected to transcriptome analysis. These cattle were from Inner Mongolia Aokesi Livestock Breeding Co., Ltd and were raised in the same feeding strategies and conditions. Slaughtering and sampling were completed in Zhongao Food Co., Ltd (Aohan Banner, Chifeng City, Inner Mongolia). Cattle stopped feeding and drinking strictly 24 h before slaughter. The LD muscle (12-13th ribs) was harvested within 30 min after slaughter and the samples were washed with phosphate-buffered saline (PBS) to avoid contaminating the muscle tissues during the operation. Afterward, pieces of LD muscle tissues were obtained and put into Eppendorf (EP) tubes. All samples were immediately frozen in liquid nitrogen for total RNA extraction. In addition, 1 kg of the LD muscle (11-13th ribs) of the left carcass per sample was collected after 24 h of acid removal at 4℃. After vacuum packing, all the LD muscles were stored at − 20℃ and transported to the Institute of Animal Science, Chinese Academy of Agricultural Sciences (CAAS) for meat traits measurements.

### Measurements of meat quality traits

The measurements of meat quality traits as follows: The WHC and the rate of 35 kg water loss were determined using TA-XT plus Texture Analyser 12785 (Stable Micro Systems Ltd, Godalming, Surrey GU7 1YL, UK) according to reference NY/T 1333-2007. Measurements for IMF were conducted by Soxhlet extraction anhydrous ether in accordance with GB 5009.6-2016. The SF was calculated following NY/T 1180-2006 method using a universal Warner–Bratzler testing machine MTS Synergie 200 (G-R Manufacturing Company, Manhattan, KS). Ultimate pH was measured directly on the surface of LD muscle at about 24 h after slaughter by using the pH meter HI 99163 (HANNA Instruments, Woonsocket, RI, USA).

### Total RNA extraction, library construction, and sequencing

Total RNA was isolated from individual LD tissue using TRIzol reagent (Invitrogen, Life Technologies) according to the protocol of instruction. The concentration, purity, and integrity of RNA were used to evaluate the total RNA quality. The RNA concentration was tested by Qubit RNA Assay Kit (Life Technologies, CA, USA), RNA purity was assessed using Nanophotometer Spectrophotometer (Thermo Fisher Scientific, MA, USA), and RNA integrity was measured through the RNA Nano 6000 Assay Kit of the Bioanalyzer 2100 system (Agilent Technologies, CA, USA). Then, high-quality samples (28S/18S > 1.8 and OD 260/280 ratio > 1.9) were used to construct cDNA libraries and applied for RNA sequencing if the RNA Integrity Number (RIN) was more than 7. The construction of cDNA libraries was generated using IlluminaTruSeqTM RNA Kit (Illumina, USA) following the manufacturer’s instructions and the RNA sequencing was performed on an Illumina NovaSeq 6000 platform by paired-end strategy (read length 150 bp). The RNA sequencing was completed by Beijing Novogene Technology Co., Ltd.

### Quality control of sequencing data

To obtain clean reads, the MD5 value was used to check the integrity of the original sequencing read. Using FastQC (v0.11.9) to evaluate the read quality in terms of base composition and quality distribution, then visualizing all sequencing results through MultiQC (v1.9). Using adaptive trimming algorithm of Trimmomatic (v0.39) tools to perform quality filtering, discarding reads containing ploy-N (the percentage of undetermined base information is greater than 5% in a read), trimming adaptors and low-quality reads. Subsequent data analysis is based on clean reads obtained through the above steps.

### Reads mapping

HISAT2 (v2.2.1) was used to compare clean reads to reference genome *Bos taurus* ARS-UCD1.2 (ftp://ftp.ensembl.org/pub/release-101/fasta/bos_taurus/dna/)^92^. Effective reads aligned to the gene region were statistically calculated according to the genomic location information specified by the cattle reference genome annotation (ftp://ftp.ensembl.org/pub/release-101/gtf/bos_taurus/). SAM files generated by the HISAT2 were sorted through SAMtools (v1.11). FeatureCounts (v1.5.2) was used to estimate read counts generated from RNA sequencing experiments^93^.

### Differentially expressed genes identification and function enrichment analysis

A total of eight individuals in the two groups with significant differences in the WHC were selected for transcriptome analysis to identify potential candidate genes affecting the WHC. Differential gene expression analysis was analyzed using DESeq2 (v1.18.0)^94^, which calculates differential expression based on the negative binomial distribution. Benjamini–Hochberg approach was used to adjust p-values for controlling the FDR. Genes with padj < 0.01 and log_2_FC ≥ 1 or log_2_FC ≤ -1 were identified as DEGs. Heatmap was drawn by pheatmap (v1.1.7) package^95^. To understand the function of DEGs, GO and KEGG pathway enrichment analyses were performed using the “clusterProfiler” based on the hypergeometric model^96^. GO terms were divided into three categories, namely, BP, CC, and MF. KEGG pathway analysis revealed the role of DEGs in metabolic pathways and specific biological functions. Those GO terms and pathways with an adjusted p-value of less than 0.05 and q-value less than 0.05 were considered to be significantly enriched. The STRING was further used to carry out PPI network analysis.

### DEGs comparison with the QTLs and previous reports affecting WHC

With the development of high-throughput sequencing technologies, the genetic mapping of QTLs has provided well-defined genetic maps for meat quality traits^97^. The Animal QTLdb is open that provides dynamic, updated publicly available trait mapping data to locate and compare discoveries within and between species. Up to now, a total of 160,659 QTLs from 1030 publications that contain 675 phenotypic traits have been collected in the current release of the Cattle QTLdb (https://www.animalgenome.org/cgi-bin/QTLdb/BT/index). In order to screen the DEGs for the candidate genes associated with beef WHC, we compared the DEGs with QTLs in the cattle QTLdb and previous reports of WHC trait. The DEGs mapping to QTL related to the WHC trait deserved further investigation and discussion.

### Statistical analysis of animal performance

Using the Independent-Sample T-test procedure and Pearson coefficient calculation of SPSS (v20.0) to assess the measurement results of meat traits. All data presented in the table were expressed as means ± standard deviation (M ± SD).

## Supplementary Information

Supplementary Table S1.

Supplementary Table S2.

Supplementary Table S3.

Supplementary Table S4.

Supplementary Table S5.

Supplementary Table S6.

Supplementary Table S7.

Supplementary Table S8.

## Data Availability

RNA-seq data has been submitted to Sequence Read Archive (SRA) with accession number SRR14209399, SRR14209400, SRR14209401, SRR14209402, SRR14209403, SRR14209404, SRR14209405, and SRR14209406. The data will be accessible with the following link on May 1, 2022: https://www.ncbi.nlm.nih.gov/sra/PRJNA721166. The following are available at supplementary materials, Supplementary Table S1 Phenotypic information of the WHC trait for the low and high samples, Supplementary Table S2 The primary information of sequencing reads alignments to Bos taurus reference genome, Supplementary Table S3 All DEGs detected between high and low WHC groups, Supplementary Table S4 GO terms significantly enriched with DEGs, Supplementary Table S5 KEGG pathways significantly enriched with DEGs, Supplementary Table S6 Comparison of DEGs with QTLs influencing WHC, Supplementary Table S7 The detailed information of candidate genes affecting WHC trait, Supplementary Table S8 The involvement of novel potential candidate genes in significantly enriched GO terms and pathways.
